# Method Validation and Dissipation Behaviour of Dimethyl Disulphide (DMDS) in Cucumber and Soil by Gas Chromatography-Tandem Mass Spectrometry

**DOI:** 10.3390/ijerph16224493

**Published:** 2019-11-14

**Authors:** Abdul Kaium, Junli Cao, Xingang Liu, Jun Xu, Fengshou Dong, Xiaohu Wu, Yongquan Zheng

**Affiliations:** 1 State Key Laboratory for Biology of Plant Disease and Insect Pests, Institute of Plant Protection, Chinese Academy of Agricultural Sciences, Beijing 100193, China; kaium.agrichemistry@gmail.com (A.K.); cjlagriculture@163.com (J.C.); xujun1977927@163.com (J.X.); fsdong@ippcaas.cn (F.D.); xhwu@ippcaas.cn (X.W.); yqzheng@ippcaas.cn (Y.Z.); 2 Department of Agricultural Chemistry, Sher-e-Bangla Agricultural University, Dhaka 1207, Bangladesh; 3 College of Chemistry, Central China Normal University, Wuhan 430079, China

**Keywords:** dimethyl disulphide (DMDS), cucumber, soil, gas chromatography-mass spectrometry

## Abstract

In this study, a useful analytical method was developed and validated for measuring the residues of dimethyl disulphide (DMDS) in cucumbers and soil by gas chromatography-tandem mass spectrometry (GC-MS/MS). The dissipation dynamics and residual levels of DMDS in cucumber and soil were also studied in Shandong, Jilin, and Hebei provinces by using this method. Dichloromethane was selected and used as the extraction solvent to extract the target pesticide from the soil and cucumber samples. The soil and cucumber samples were cleaned up by the combination of multiwalled carbon nanotubes (MWCNT) and biochar. The average recoveries of the DMDS in cucumbers and soil were in the range of 84–101.5%, with relative standard deviations (RSD) of 0.7–4.9%, when they spiked at 0.05, 0.5, and 5 mg/kg DMDS respectively. The limit of quantification (LOQ) of this method was 0.05 mg/kg. First-order and second-order kinetic equations were used to fit dissipation data. Results show that the half-lives of DMDS in the soil at Shandong, Jilin, and Hebei were 1.63–4.47 days, 1.96–6.49 days, and 1.35–2.51 days, respectively. The final residues of DMDS were less than 0.05 mg/kg in cucumbers and 0.36 mg/kg in the soil. The dissipation rates of DMDS in different soils were different. The method provides a basis for the risk assessment of DMDS in cucumber and soil.

## 1. Introduction

**Figure 1:**
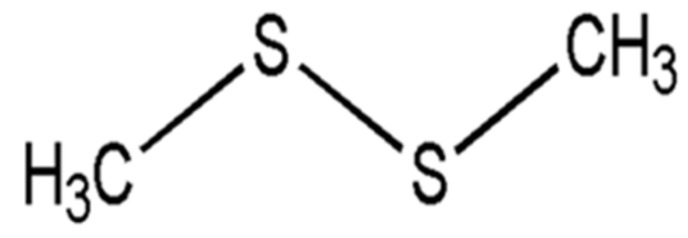
The chemical structure of dimethyl disulphide (DMDS).

Soil-borne disease and pests are becoming a significant issue in vegetable production worldwide [1]. In the vegetable production system, chemical fumigants have been used to control soil-borne disease and pests for a long time [2]. Methyl bromide (MeBr) was the only chemical fumigant that was used extensively in the past 40 years to control soil-borne disease and pest in agriculture [3,4]. MeBr was phased out worldwide in 2015 due to its stratospheric ozone depletion potential [4,5]. As a substitute for MeBr [6,7], dimethyl disulphide (DMDS, see Figure 1)—a volatile sulfur compound that has zero ozone depletion potential—was first registered and applied in the United States as a plant pre-pest management soil fumigant [8]. Since 2001, DMDS has been one of the most experimented and discussed volatile chemical compounds for soil disinfestation in the US, EU, China, and the Mediterranean countries [9,10,11,12,13]. Nowadays, it has been widely used to protect vegetables all over the world. Several trials of DMDS efficiency have shown excellent control of broad-spectrum soil-borne pests like nematode, fungus, insects, and weeds [10,12,14,15,16,17]. For best performance, the fumigant must move across the soil profile and last an appropriately long time to ensure successful soil pest control [18,19]. Conkle et al. [20] reported that DMDS might need to be applied at a high dose to achieve higher efficacy due to its lower volatility and higher soil adsorption rate. However, soils with high fumigant concentrations can cause phytotoxicity, which usually results in low yields or even plant death. Therefore, it is necessary to establish an effective detection method to study the degradation dynamics and residual behavior of DMDS in soil and crops.

There are some techniques for determining DMDS residues in the soil, including Fourier transform infrared spectroscopy (FTIR) [21], gas chromatography equipped with flame ionization detector (GC-FID) [22] and gas chromatography-mass spectrometry (GC-MS) [23]. However, to the authors’ knowledge, there is no report of a method for the determination of DMDS residue in cucumber samples. Therefore, minimal data have been reported concerning the dissipation of DMDS in cucumber and soil under open field conditions in the different growing areas of China.

In this study, an efficient analytical method using gas chromatography coupled with tandem mass spectrometry was proposed to determine the DMDS residues in cucumber and soil. Moreover, the dissipation rate and final residue level of DMDS under open field conditions were studied in different growing areas of China. The residual dissipation and final residue of DMDS were determined to provide evaluation for the safe use of DMDS.

## 2. Materials and Methods

### 2.1. Standards and Reagents

DMDS (purity 99.6%) was provided by French company Arkema. HPLC-grade ethyl acetate, cyclohexane, acetone, petroleum ether, and dichloromethane were bought from the Beijing Chemical Company (Beijing, China). Graphitized carbon black (GCB, 120–400 mesh), multi-walled carbon nanotubes (MWCNT, 20–30 µm) were purchased from Bonna-Agela Technologies (Tianjin, China) and Biochar (200–400 mesh) was from Panzhihua Xiyu Biological Technology Co. Ltd. (Panzhihua, China). The standard stock solutions of DMDS were prepared in pure dichloromethane. Standard working solutions of 0.05, 0.1, 0.2, 0.5, 1, 2 and 5 mg/L were prepared by serial diluting from the stock solution. The matrix-matched standard solutions were prepared (0.05, 0.1, 0.5, 1, 2 and 5 mg/L) by adding a blank matrix (cucumber and soil) to each serially diluted standard solution. All solutions were stored in a freezer at −20 °C, ready for use.

### 2.2. Field Study

The cucumber field experiments were conducted in Shandong, Jilin, and Hebei province of China from September 2015 to October 2016. The atmospheric and soil physicochemical parameters of these three experimental sites are listed in Table 1. According to the pesticide residue standard requirement test, each treatment consisted of three replicates and every plot with an area of 3 × 5 m (15 m^2^) [24]. For the dissipation studies, 99.6% pure DMDS was applied once to the cucumber field soils by syringe with a dosage 90 g/m^2^ 1.5 times than the recommended dosage [8]. The soils were covered with a protective film after the application of DMDS. Cucumber samples (approximately 500 g) were collected from each plot after spraying on days 0 (2, 4, 8, 12 h), 1, 3, 5, 7, 14, 21, 28 days, respectively, while soil samples (0–15 cm depth, approximately 500 g) were randomly collected at 0 (2, 4, 8, 12 h), 1, 3, 5, 7, 14, 21, 28 days respectively. All the collected samples were stored in a freezer at −20 °C until extraction. For the final residue experiment, DMDS was applied at 60 g/m^2^ and 90 g/m^2^ in each experimental cucumber field. Each dosage level was designed to be applied once before seed sowing. Therefore, cucumber and soils (0–15 cm depth) samples were collected at intervals of 0, 1, 2, 3, 5 and 7 days after cucumber maturation. All the samples were transported in labeled polythene bags and stored at −20 °C for further analysis. 

### 2.3. Sample Preparation Procedure

Cucumber and soil samples were blended separately by a homogenizer, and 5 g of each sample was transferred to a 20 mL headspace vials, then added 5 mL dichloromethane, 3 g sodium chloride and purification agents (25 mg MWCNT + 50 mg Biochar) to each vial. These vials were sealed with a Teflon film aluminium cover and placed in a water bath with static 80 °C temperatures for 30 min. After cooling, a 1 mL lower layer of dichloromethane extract was taken with a syringe, then filtered through a 0.22 µm PTFE filter and transferred into a glass vial for GC-MS/MS analysis.

### 2.4. Instrumental and Analytical Conditions

A Varian 450GC–300MS was used to detect and analyze the samples. The chromatographic separation was performed on a DB-5MS analytical column (30 m × 0.25 mm, i.d. × 0.25 μm). Helium (99.9999%) was used as the carrier gas at the flow rate of 1.0 mL/min. The temperature of the injector port was 230 °C, and a volume of 5 μL was injected in the splitless mode. The samples were analyzed using the oven temperature program: Initial temperatures of 40 °C (hold for 3 min), then ramped up to 50 °C at 5 °C/min (hold for 7 min). The ion source and transfer line temperatures were set at 230 °C. The mass spectrometer was operated in electron ionisation mode at 70 eV. Detection was performed in the 79 *m*/*z*, 61 *m*/*z*, and 45 *m*/*z* selected ion monitoring (SIM) mode for the quantitative and qualitative analysis.

### 2.5. Method Validation

According to European Union guidelines (SANTE/11813/2017) [25], the method was validated on parameters of selectivity, linearity, accuracy, precision, the limit of detection (LOD) and limit of quantification (LOQ). Linearity was determined by constructing calibration curves using standard solutions of different concentrations (0.05–5 mg/kg). The LOD was calculated as the lowest concentration giving a response three times the standard deviation of the baseline noise. The LOQ was calculated as the lowest spiked level during the recovery experiments that provide satisfactory values of recovery (70–120%), and relative standard deviation (RSD) ≤20%. Recovery and repeatability (RSD) experiments were carried out to investigate the accuracy and precision of the method at three levels of fortification 0.05, 0.5 and 5 mg/kg DMDS in cucumber and soil replicated five times alongside a control. In this method, the accuracy was determined by average recovery assays obtained according to the standard European Union guidelines, and the precision was determined by the relative standard deviation (RSD).

### 2.6. Statistical Analysis

The first-order kinetics model and the second-order kinetics model were used to describe DMDS dissipation. The degradation rate constants and half-lives were calculated using equations C_t_ = C_0_ e^−kt^ and C_t_ = C_0_/(1 + C_0_ × kt), where C_t_ represents the concentration of DMDS residue at the time of t, C_0_ represents the initial concentration, and k is the dissipation degradation rate constant (days − 1). T_1/2_ represents the time when the residual is half of the initial concentration. All the calculations were made by Microsoft Office Excel 2010 software. 

## 3. Results and Discussion

### 3.1. Validation of the Method

#### 3.1.1. Matrix Effects, Linearity, LODs and LOQs

The proposed method was applied to the blank samples of different matrices to evaluate its specificity. The calibration curves for DMDS (from 50 to 5000 mg/kg) in dichloromethane and different matrices were obtained, and the linear regression results were summarized in Table 2. The results showed that the cucumber matrix significantly suppressed the instrument’s response, and no significant suppression or improvement of DMDS response was observed in the soil matrix. The response of standard solution calibration curves was compared to matrix-matched calibration curves using a two-tailed paired *t*-test with a probability of 95%. The *P* values imply that the data between pure solvent and cucumber are significantly different (*P* < 0.05) and have no significant difference (*P* > 0.05) with soil. Therefore, matrix-matched standards were used to eliminate matrix effects.

**Figure 2:**
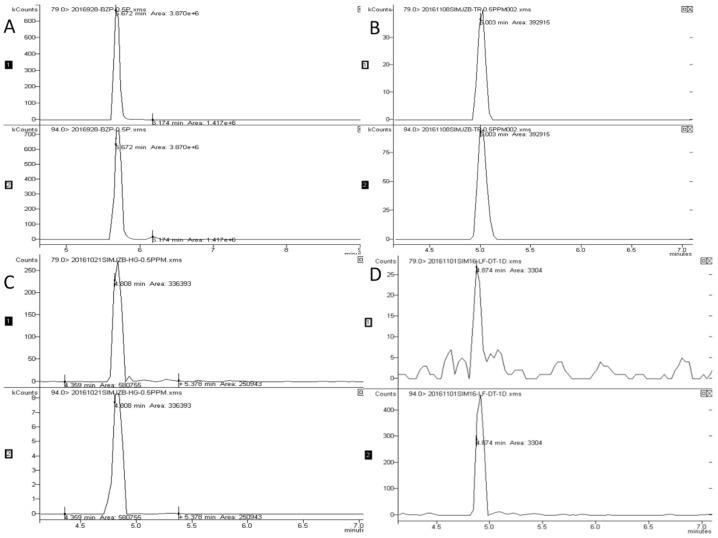
Chromatograms of the (**A**) DMDS standard, (**B**) soil spiked at 0.5 mg/kg, (**C**) cucumber spiked at 0.5 mg/kg, (**D**) DMDS residue in soil on the 1st day after application in the Hebei location.

DMDS was detected at the retention time of 5.0 min (Figure 2). The linearity, LOD, and LOQ were obtained using the peak areas of the product ion obtained by GC-MS analysis using the SIM mode. The results showed that the linearity is excellent, with a regression correlation coefficient of R^2^ = 0.9996. The LOD and LOQ were determined as 0.015 and 0.05 mg/kg.

#### 3.1.2. Optimization of the Extraction Method

**Figure 3:**
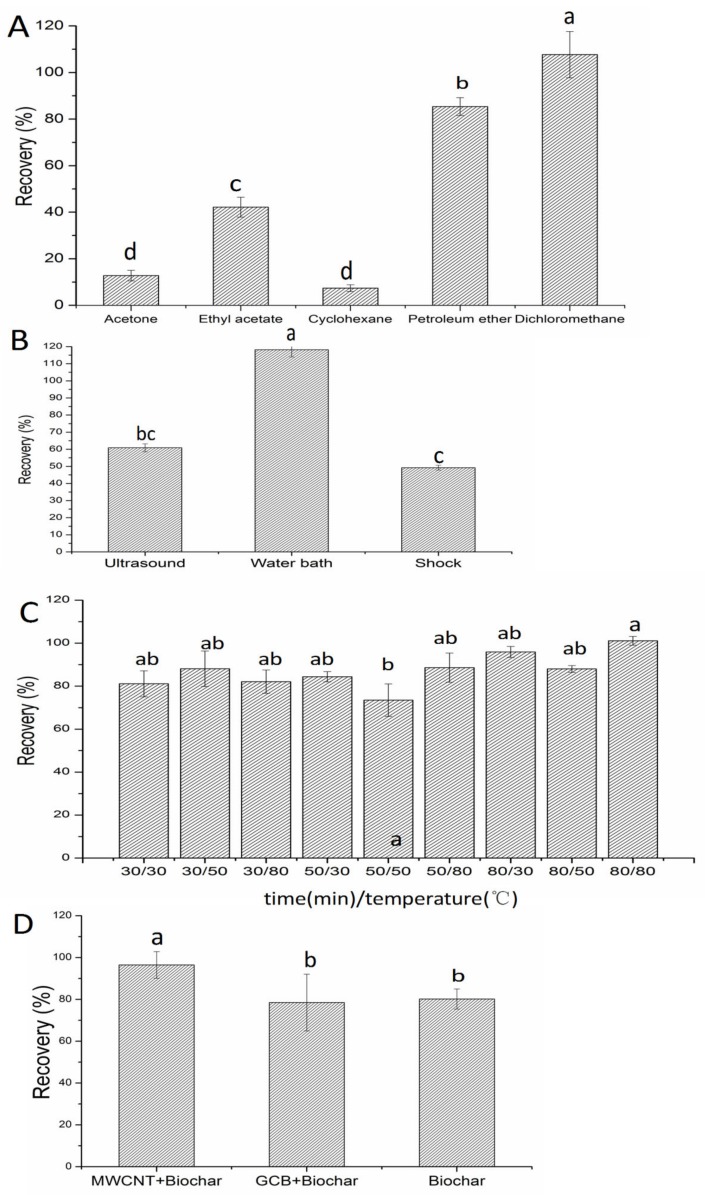
Comparison of recoveries of DMDS in soil samples (0.5 mg/kg) with (**A**) different extraction solvents, (**B**) different extraction methods, (**C**) different water bath time and temperature, (**D**) different cleanup sorbents.

In this work, three extraction methods in brown soil were compared: Shock extraction, water bath, and ultrasound extraction for 20 min. The results in Figure 3 showed that the water bath offered higher recoveries (118.2%) than ultrasound extraction (60.9%) and shock extraction (49.2%). For the residue determination method, recoveries in the range of 70% to 120% are considered acceptable. Therefore, the water bath was chosen as the extraction method. Five organic solvents (acetone, ethyl acetate, cyclohexane, petroleum ether, dichloromethane) were then compared to the solvent with the highest extraction efficiency. As shown in Figure 3, the recovery of DMDS extracted with dichloromethane (107.7%) was higher than acetone (12.8%), ethyl acetate (42.2%), cyclohexane (7.4%) and petroleum ether (85.4%). Therefore, dichloromethane was selected as the extractant for the following experiments. However, different water bath times and temperatures could affect the recoveries. This is because short time makes extraction insufficient while delaying time, and whether the temperature is too high or too low will reduce the extraction efficiency. As shown in Figure 3, when the temperature was 80 °C, and the bath time was 30 min and 80 min, the recoveries were 95.9% and 101.1%. In view of the efficiency of the experiment, 80 °C water bath for 30 min was chosen as the extraction technique for this method.

In order to achieve a satisfactory clean-up effect, three types of sorbent, biochar (50 mg) + MWCNT (25 mg), biochar (50 mg) + GCB (25 mg) and biochar (75 mg) were evaluated in this work to investigate the impacts on recoveries in cucumber and soil matrices. GCB is mainly used to remove pigments such as chlorophyll and carotenoids [26]. MWCNT has been reported as a new type of purification material with a better purification effect for its larger specific surface area [27]. Biochar has commonly been used in water and soil cleanup and toxic material remediation from the environment [28]. However, on the other site, biochar has a very high affinity and capacity to sorbing organic compounds [29]. In this experiment, biochar was for the first time used as a cleanup sorbent for pesticide residue extraction from the soil and cucumber samples. The results show that when biochar with a certain proportion of GCB was used as a cleanup sorbent in soil matrices, the recoveries of DMDS were relatively low (78.5%), which may mean that the interfering impurity was not completely eliminated in this matrix, but that the biochar with MWCNT gave a higher recovery (96.5%). Finally, the biochar (50 mg) + MWCNT (25 mg) was selected as the final cleanup sorbent to clean up impurities in soil and cucumber matrices.

#### 3.1.3. Accuracy and Precision

The accuracy and precision of the measurements were expressed in terms of recoveries and RSDs at different spiked levels (0.05, 0.5 and 5 mg/kg) in cucumber and soil. DMDS recoveries for cucumber and soil ranged between 84.0–101.5% and 84.3–98.7%, respectively, with RSDs between 0.5–6.5% and 0.7–4.9% (Table 3). The results have confirmed that the method is sufficiently reliable for DMDS residue analysis in this experiment [30].

### 3.2. Dissipation Dynamics

The first-order kinetic equation is commonly used to describe pesticide dissipation dynamics. However, the first-order equation does not take into consideration the environmental effects of these dissipation studies, which are supported by Gottschalk et al. [31]. In this study, the first-order kinetic equation and the second-order kinetic equation both were used to fit the dynamic data simultaneously.

#### 3.2.1. Dissipation of DMDS in Soil

**Figure 4:**
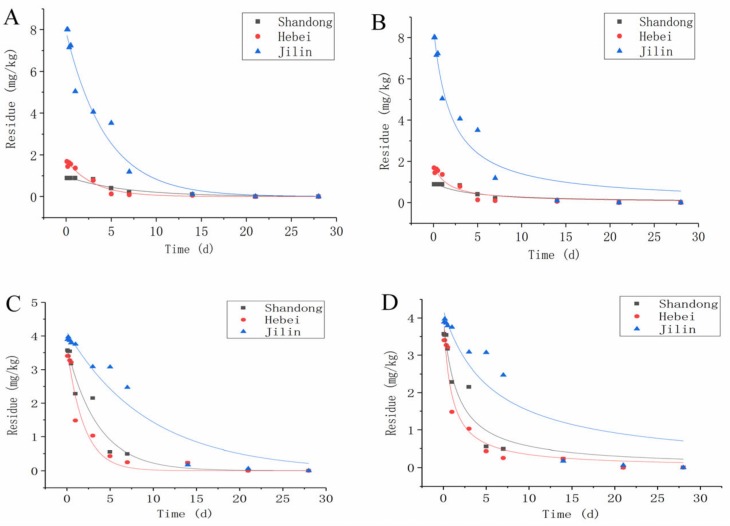
Dissipation dynamics of DMDS in the soil of three different experimental sites of China: (**A**) according to the first-order kinetic equation in 2015, (**B**) according to the second-order kinetic equation in 2015, (**C**) according to the first-order kinetic equation in 2016, (**D**) according to the second-order kinetic equation in 2016.

The dynamics of dissipation and half-life of DMDS in soil are presented in Figure 4 and Table 4. In order to interpret the dissipation of the DMDS in soil, other regression models were adapted to total mass, although pesticide dissipation in the soil is often assumed to be a first-order reaction [32,33]. R^2^ was used for comparison of model fits (Table 4). Initial DMDS concentrations in soil were 0.89 mg/kg. According to first-order kinetic equation data, the half-life degradation of DMDS in soil was 4.47, 3.07, and 2.10 days for 2015 in the provinces of Shandong, Jilin, and Hebei, while the half-life was 2.49, 6.49 and 1.35 days for 2016. According to the second-order kinetic equation for 2015 and 2016, the DMDS dissipated with a half-life of 3.70–1.63 days, 1.96–5.56 days, and 1.55–2.51 days in Shandong, Jilin, and Hebei, respectively. The second-order equation also supports the first order dissipation regression equation.

Figure 4 shows the residue of DMDS in soil over the 2015 and 2016 test period in three different provinces in China. The curves of DMDS residue degradation dynamics in Shandong, Jilin and Hebei soil also had good comparability in 2 years and could be calculated by both the first-order kinetic equation and second-order kinetic equation. The results showed that the residue of DMDS was quickly dissipated in cucumber field soil. As expected, gradual and continuous degradation of the DMDS residues in the cucumber fields was observed, depending on the time after application. In the first 14 days after application, a rapid decrease in the amount of DMDS residues occurred and dissipated more than 90% after 14 days. DMDS dissipates faster in Hebei, is intermediate in Jilin and is slowest in Shandong because environmental and soil physio-chemical characteristics are different in Shandong, Jilin, and Hebei. It is reported that pesticide dissipation was a complex process depending on various environmental and soil physiochemical factors such as rainfall, temperature, relative humidity, soil organic matter, and soil pH [34].

#### 3.2.2. Dissipation of DMDS in Cucumber

The dissipation study is an important part and would be useful for the proper and safe use of pesticides. A modified GC-MS/MS method was developed and used in this experiment to detect DMDS residues in samples collected from the provinces of Shandong, Jilin, and Hebei from September 2015 to October 2016 to investigate dissipation kinetics. We found a total of less than 0.05 mg/kg DMDS residue in cucumber samples collected after DMDS application, which is about less than DMDS LOQ in this method, so no further dissipation studies are required.

#### 3.2.3. The Final Residue of DMDS in Cucumber and Soil

For cucumbers, the final residue of DMDS was <0.05 mg/kg, and for soil, the final residue was less than 0.36 mg/kg. In this experiment, the residue of DMDS was <0.05 mg/kg before the cucumber matured. The maximum residue limit (MRL) of DMDS in cucumber has not been established in China, the EU, the UK, and Japan. This study can provide basic data for the proposed DMDS MRL.

## 4. Conclusions

The GC-MS/MS residue analytical method has been validated and used for the determination of DMDS residues in cucumber and soil samples. By using this method, the experiment results showed that DMDS was susceptible to dissipate with half-lives ranging from 1.35 to 6.49 days in soil under the field ecosystem. The dissipation rates vary between the experimental sites of Shandong, Jilin, and Hebei, suggesting that dissipation of DMDS depends on environmental and soil physiochemical factors. The results showed that DMDS decreased very rapidly in open field conditions and finally dissipated over 90% within 14 days of application. The final residues of DMDS in soil and cucumber were 0.36 and <0.05 mg/kg, respectively, which is much lower than DMDS LOQ. These results showed that cucumbers could be safe when applied at the recommended dosage and suggested that the maximum residue limit of DMDS in cucumber is 0.05 mg/kg.

## Figures and Tables

**Table 1:** Atmospheric and soil physicochemical parameters of three experimental field sites.

Experimental Sites	Climate	Average Annual Rainfall (mm)	Average Annual Temperature (°C)	Soil Type	Organic-Matter (%)	Soil pH
Shandong	semi-humid monsoon	900–750	13	Brown soil	2	7.15
Jilin	temperate monsoon	400–600	17	Sandy loam	3.40	7.3
Hebei	temperate monsoon	400–800	11	Clay	1.80	6.5

**Table 2:** Calibration equation and linear regression parameters of calibration curves of DMDS in solvent and matrices.

Matrix	Calibration Equation	Coefficient (*R*^2^)	*P* Value	LOD (mg/kg)	LOQ (mg/kg)
Dichloromethane	*y* = 8656123 *x* + 719091	0.9996	-	0.012	0.05
Cucumber	*y* = 6808925 *x* − 936245	0.9956	0.041	0.015	0.05
Soil	*y* = 7811030 *x* + 1803515	0.9953	0.49	0.015	0.05

**Table 3:** Average recoveries and relative standard deviations (RSD) of cucumber and soil samples (n = 5).

Matrix	Spiking 0.05 mg/kg		Spiking 0.5 mg/kg		Spiking 5 mg/kg	
	Mean Recovery (%)	RSD (%)	Mean Recovery (%)	RSD (%)	Mean Recovery (%)	RSD (%)
Cucumber	83.98	6.5	90.28	0.5	101.51	3.8
Soil	74.07	4.9	84.33	0.7	98.69	4.8

**Table 4:** The regression equation and half-life for DMDS dissipated in soil.

Time (year)	Locality	First-Order Kinetic Equation			Second-Order Kinetic Equation		
		Regression Equation	Coefficient (R^2^)	Half-Life (day)	Regression Equation	Coefficient (R^2^)	Half-Life (day)
2015	Shandong	y = 0.96249e^−0.15433x^	0.9498	4.47	y = 0.98888/(1 + 0.2702x)	0.8943	3.70
	Jilin	y = 7.86281e^−0.22474x^	0.9720	3.07	y = 8.5319/(1 + 0.5096x)	0.958	1.96
	Hebei	y = 1.74864e^−0.32911x^	0.9757	2.10	y = 1.8531/(1 + 0.6463x)	0.9334	1.55
2016	Shandong	y = 3.64191e^−0.27719x^	0.9628	2.49	y = 3.9897/(1 + 0.614x)	0.9441	1.63
	Jilin	y = 4.1209e^−0.10624x^	0.9430	6.49	y = 4.20337/(1 + 0.1799x)	0.8679	5.56
	Hebei	y = 3.66312e^−0.51003x^	0.9618	1.35	y = 4.05637/(1 + 0.3984x)	0.9604	2.51
